# High-fat diet impacts more changes in beta-cell compared to alpha-cell transcriptome

**DOI:** 10.1371/journal.pone.0213299

**Published:** 2019-03-08

**Authors:** Rodolphe Dusaulcy, Sandra Handgraaf, Florian Visentin, Cedric Howald, Emmanouil T. Dermitzakis, Jacques Philippe, Yvan Gosmain

**Affiliations:** 1 Molecular Diabetes Laboratory, Division of Endocrinology, Diabetes, Hypertension and Nutrition, University Hospital/Diabetes Center/University of Geneva Medical School, Geneva, Switzerland; 2 Department of Genetic Medicine and Development, University of Geneva Medical School, Geneva, Switzerland; Case Western Reserve University School of Medicine, UNITED STATES

## Abstract

Characterization of endocrine-cell functions and associated molecular signatures in diabetes is crucial to better understand why and by which mechanisms alpha and beta cells cause and perpetuate metabolic abnormalities. The now recognized role of glucagon in diabetes control is a major incentive to have a better understanding of dysfunctional alpha cells. To characterize molecular alterations of alpha cells in diabetes, we analyzed alpha-cell transcriptome from control and diabetic mice using diet-induced obesity model. To this aim, we quantified the expression levels of total mRNAs from sorted alpha and beta cells of low-fat and high-fat diet-treated mice through RNAseq experiments, using a transgenic mouse strain allowing collections of pancreatic alpha- and beta-cells after 16 weeks of diet. We now report that pancreatic alpha cells from obese hyperglycemic mice displayed minor variations of their transcriptome compared to controls. Depending on analyses, we identified 11 to 39 differentially expressed genes including non-alpha cell markers mainly due to minor cell contamination during purification process. From these analyses, we identified three new target genes altered in diabetic alpha cells and potently involved in cellular stress and exocytosis (*Upk3a*, *Adcy1* and *Dpp6*). By contrast, analysis of the beta-cell transcriptome from control and diabetic mice revealed major alterations of specific genes coding for proteins involved in proliferation and secretion. We conclude that alpha cell transcriptome is less reactive to HFD diet compared to beta cells and display adaptations to cellular stress and exocytosis.

## Introduction

Obesity is associated with insulin resistance and an increased type 2 diabetes (T2D) risk [1]. When insulin resistance is accompanied by dysfunction of pancreatic islet cells, hyperglycemia results [2]. The disrupted coordination of glucagon and insulin secretion observed in type 2 diabetes is characterized by impaired and delayed insulin secretion as well as basal hyperglucagonemia and non-suppressed glucagon secretion in response to glucose [3, 4]. A large number of studies have examined the consequences of diabetes on pancreatic islets using different animal models among them diet-induced obese mice (DIO) [5]. High-fat diet (HFD) fed mice exhibit impaired glucose tolerance and insulin resistance leading to hyperglycemia, hyperinsulinemia and dysregulated glucagon secretion [6]. Glucagon, produced by pancreatic alpha-cells, shows dysregulated secretion in both type 1 and type 2 diabetes and contributes to hyperglycemia [3, 7–10]. The consequences of increased glucagon during fasting and unsuppressed glucagon secretion in response to meals are an increased rate of hepatic glucose production contributing to hyperglycemia.

Therapies directed at blocking glucagon action clearly improve glycemic control in both type 1 and type 2 diabetic patients as well as in rodent diabetic models. Indeed, administration of the glucagon receptor antagonist LY2409021 lowers blood glucose levels in T2D patients highlighting the interest to block glucagon action in diabetes [11]. In mice, knockout of the glucagon receptor gene led to reduced plasma glucose levels, improved glucose tolerance and refractoriness to the development of hepatic steatosis [6]. In addition, these mice are more resistant to streptozotocin (STZ)-induced hyperglycemia and pancreatic β-cell destruction [6, 12]. Furthermore, glucagon receptor antagonists or glucagon antibodies in diabetic mice clearly improve glycemia, suggesting that glucagon represents a critical component of hyperglycemia in induced diabetic models [13, 14].

These observations demonstrated that T2D pathophysiology should be attributed both to an excess of glucagon and insulin deficiency implicating glucagon-producing alpha and insulin-producing beta-cell dysfunction.

This, now recognized, role of glucagon in diabetes control is a major incentive to have a better understanding of alpha cells dysfunctions.

Alpha cells are not only producing glucagon but are also an intra-islet source of glucagon-like peptide 1 (GLP-1) since the prohormone convertase PC1 is detected at least in a portion of cells both in humans and rodents [15, 16]. Indeed, GLP-1 production and PC1 expression by alpha-cells are increased in specific situations such as obesity and diabetes [15–20]. As previously proposed, these observations may represent an adaptive response to hyperglycemia and a local answer to beta-cell dysfunctions and death [21]. Furthermore, alpha cells may represent a potential source of insulin-producing cells in regenerative models [22, 23]. Following these observations, alpha cells represent an emerging way to improve beta-cell function and glycemia in diabetes. These results indicate that changes in alpha-cell function are much wider than dysregulated secretion of glucagon.

We previously described the moderate alterations of the expression of a limited number of genes involved in the function of alpha cells from fat-induced diabetic mice by candidate gene analysis [24]. Analyses of alpha-cell transcriptome by cDNA array or RNA sequencing would be an important addition to our results as it would come from an unbiased and more global investigation.

Single-cell transcriptome profiling studies have been developed using sorted pancreatic cells from diabetic patients and have opened the way to exhaustive molecular characterizations [25, 26]. Despite improvements in these advanced technologies, unreproducible results have been generated probably secondary to biological and technical limitations such as differences in patient characteristics, islet isolation procedures, time lags between death and cell isolation as well as analytical methodologies especially for low sample size data. Thus, molecular characterizations of alpha and beta cells in obesity and diabetes remain necessary for a better understanding of pancreatic endocrine-cell function and potential designs of therapeutic approaches for diabetes care.

We thus analysed the expression of polyA+ RNA of sorted alpha and beta cells from DIO mice. To this end, we used a transgenic mouse strain allowing the collection of pancreatic alpha- and beta-cell fractions from control low-fat diet (LFD) and diabetic HFD mice using cell sorters.

We now report that HFD induces higher quantitative changes in gene expression in pancreatic beta cells compared to alpha cells. Indeed, we identified only 11 to 39 genes whose expression was significantly modified in alpha-cells and 99 to 3192 in beta cells depending on analyses and methods. Moreover, real-time PCR and immunofluorescence experiments indicated that some of the genes whose expression was found to be altered between control and obese mice were due to contamination in sorted alpha cells. Finally, only three genes (*Upk3a*, *Dpp6 and Adcy1*) were validated to be differentially expressed between alpha cells of control and obese hyperglycemic mice. By contrast, beta-cell transcriptome of diabetic mice exhibited major alterations of specific genes coding for proteins involved in proliferation, secretion and ion channel activity. These minor variations of alpha cells transcriptome in obese hyperglycemic mice, compared to beta cells, suggest that alpha cell function is much less affected than beta cells function in this condition.

## Materials and methods

### Animals

The transgenic mice C57Bl/6J-Tg(GLU-Venus x INS-Cherry) express specifically the Venus and mCherry fluorochromes under glucagon (*Gcg*) and insulin (*Ins2*) gene promoters respectively. Animals were bred in conventional housing with a 12h/12h light/dark cycle and subjected to experimental procedures according to ethical approbation by the Swiss federal committee in the University of Geneva Medical School. The protocol was approved by the Swiss federal committee (approval number GE/143/18). 10 to 12 weeks-old male GLU-Venus x INS-Cherry mice [27] were fed ad libitum with a low-fat diet (LFD-control, 10% of fat) or a high-fat diet (HFD, 60% of fat) during 16 weeks. Littermate animals were randomly included in LFD and HFD groups. In each experiment, we compared obese hyperglycemic HFD mice to the control LFD group. Weight and glycated hemoglobin (HbA1c) were evaluated from LFD and HFD mice after 16 weeks of diet as described (S1 Table) [24]. Mice were anesthetized by ketamine/xylazine injection and killed by exsanguination during surgery protocol.

### Primary cell sorting, RNA extraction, library preparation, sequencing, mapping and expression quantification

After 16 weeks of diet, primary Venus+ alpha and Cherry+ beta cells from LFD and HFD mice were separated from other pancreatic cells by fluorescence-activated cell sorting (FACS) using Biorad S3 or Astrios (S1 Fig) after standard isolation procedures on pancreas as described [28].

Total mRNA, extracted from Venus+ alpha and Cherry+ beta cells of LFD and HFD mice, was prepared using the RNeasy plus micro kit (Qiagen, Hilden, Germany). PolyA+ RNA library construction and sequencing were performed as described elsewhere [29].

RNAseq libraries were generated using the Illumina TruSeq v2 protocol starting with 50ng of RNA. The quality of the libraries was assessed using a TapeStation (Agilent Technologies) and Qubit (Invitrogen). Libraries were sequenced on an Illumina HiSeq2000 producing 49bp paired-end reads. Alpha and beta samples were sequenced on an average depth of 149 and 35 millions of reads, respectively. Reads were mapped using GEMtools v1.7.1 (http://gemtools.github.io/) on GRCm38 using Gencode M4 as gene annotation. Gene quantification and normalization (RPKM) were done with QTLtools quan (doi: https://doi.org/10.1101/068635) filtering out reads with a mapping quality below 150 and paired-reads with more than five mismatches.

The differential expression analyses were performed on 4 control LFD or 4 hyperglycemic HFD samples. Samples contain pools of 2 to 4 mice to obtain 50ng of mRNA corresponding to at least 50’000 sorted Venus+ alpha and Cherry+ beta cells. Differential gene expression analyses (DGEA) were performed with DESeq2 [30] and EdgeR [31] using the average read GC content of the libraries and the RNA integrity number (RIN) as covariates. Only genes expressed at least 1 RPKM in at least 90% of the samples in one or both tested conditions were kept for the DGEA. We used multiple testing, where p-values were adjusted by the Benjamini-Hochberg which controls for false discovery rate (FDR). An adjusted p-value threshold of 0.05 was used to select differentially expressed genes (DEG). Gene enrichment analyses were performed with the web tool Webgestalt 2017 (Ref doi: 10.1093/nar/gkx356) using the full set of tested genes as reference and the DEG as the gene list. Enriched GO terms and KEGG pathways were selected using a FDR threshold of 0.05.

### Target gene analysis through real-time quantitative PCR

Total mRNA, extracted from sorted-Venus+ pancreatic alpha-cells of 6 individual mice fed a LFD and 5 individual mice fed a HFD during 16 weeks, was prepared with RNeasy plus micro kit (Qiagen, Hilden, Germany). After reverse transcription (Prime-script RT Reagent, Takara Bio Inc., Otsu, Japan), specific cDNA levels were analyzed by real-time quantitative PCR using Light-Cycler 480 SYBR Green technology (Roche Diagnostics) as described [24]. The analyses were performed using the Light-Cycler software and target gene levels were expressed relative to three reference genes (*Rps9*, *Hprt* and *Ppia*).

Data are presented as means ± SEM and analyzed using one-tailed unpaired t test with Welch’s correction for comparison between two groups in one condition. Data are statistically significant at p<0.05.

## Results

### High-fat feeding induces more quantitative changes in gene expression in beta cells compared to alpha cells

To characterize the molecular alterations of pancreatic alpha cells in diabetes; we generated DIO hyperglycemic mice using GLU-Venus x INS-Cherry transgenic mice. We analyzed and compared pancreatic alpha/beta cell transcriptomes from control normoglycemic LFD and hyperglycemic obese HFD mice selected by HbA1c values (≥ 4.5% as described) [24].

After 16 weeks of diet, we collected pancreatic alpha and beta cells from LFD and HFD mice to evaluate their transcriptomes through RNAseq analysis using four samples of both control LFD and obese HFD mice (raw data: ENA accession number PRJEB30761), and analyzed for differential gene expression using DESeq2 and EdgeR methods (S1–S5 Files). We first observed that specific markers of alpha cells, among which the *Gcg*, *Arx* and *Irx1* genes, were highly enriched in alpha-cells compared to beta cells as previously reported in human and rodent arrays [32, 33]. Similarly, beta-cell markers *Ins2*, *Ppp1r1a*, *Ucn3* and *Bace2* were highly expressed in beta cells compared to alpha cells (S2 Table). These results reflect a high enrichment of alpha and beta cells in our sorted cell fractions and thus validate our methodology.

Differential expression analyses between HFD and LFD mice from RNAseq data with the DESeq2 method revealed only 11 genes differentially expressed in Venus+ alpha cells (Table 1), including non-alpha cell genes (*Krt19*, *Sst*, *Hhex*, *Plvap*, *Iapp* and *Bace2*) as well as genes coding for proteins involved in exocytosis (*Syt5* and *Dpp6*) and in cellular stress (*Mgst1* and *8430408G22Rik/C10orf10*). By contrast, 3192 genes were significantly differentially expressed in Cherry+ beta cells of HFD hyperglycemic compared to LFD normoglycemic mice (Table 2). Among the 10 most altered genes, we identified a majority of genes coding for proteins involved in insulin secretion suggesting alterations of regulated insulin secretion. *Gc*, *Serpina7* and *Rgs4* mRNA levels were significantly upregulated in beta cells from HFD mice compared to control LFD whereas *T2*, *Trpm5* and *Kcnj12* gene expressions were downregulated. Our results on sorted beta cells from obese hyperglycemic mice are similar to a previous study directed to the effects of HFD on mouse islets [34]. Our analyses thus indicate that beta cells are quantitatively much more affected by high-fat diet compared to alpha cells.

**Table 1:** Differential gene expression analysis between HFD and LFD mice for pancreatic alpha cells using DESeq2.

Gene	Fold change	Adjusted_p-value
8430408G22Rik	1.5	7.04E-04
Hhex	0.67	7.04E-04
Krt19	1.41	1.23E-03
Mgst1	1.47	1.23E-03
Plvap	1.38	2.03E-03
Sst	0.69	2.03E-03
Dpp6	0.71	9.32E-03
Iapp	1.41	9.32E-03
Bace2	1.36	3.22E-02
Igfbp4	1.36	4.13E-02
Syt5	1.37	4.13E-02

After islet isolation, pancreatic endocrine cell dissociations and FACS sorting, mRNA levels from LFD and HFD Venus+ alpha cells were evaluated by RNAseq and analysed using the DESeq2 method (Covariates: GC mean, RIN; Adjusted p value ≤ 0.05). The fold change values represent the ratio of the results between HFD and LFD group (4 samples each). The regulated genes are listed following statistical p-values.

**Table 2:** Differential gene expression analysis between HFD and LFD mice for pancreatic beta cells using DESeq2.

Gene	Fold change	Adjusted p-value
Gc	7.83	1.33E-65
Pappa2	6.32	1.05E-36
Rgs4	5.61	1.05E-36
Serpina7	6.72	4.74E-29
Kcnj12	0.17	5.43E-25
Cd200	3.25	5.23E-22
T2	0.18	6.28E-21
Sst	0.19	2.28E-19
Cd44	3.68	6.80E-19
Trpm5	0.34	1.16E-16
…	…	…

mRNA levels from LFD and HFD Cherry+ beta cells were evaluated with RNA sequencing using DESeq2 analyses (Covariates: GC mean, RIN; Adjusted p value ≤ 0.05). Only the 10 most significant differentially expressed genes are listed (among 3192 differentially expressed genes). The fold change values represent the ratio of the results obtained between HFD to LFD group. The regulated genes are listed following statistical p-values.

### Differential results with DESeq2 and EdgeR analyses

In order to minimize false positives for low level expressed genes, DESeq2 used a transformation called logarithm fold change shrinking which shifts the fold change towards 0 for genes and conditions poorly supported. The variance of the log2 fold change is affected by the level of expression but also by the low number of reads in one of the two groups, the large variance inside a group, the number of replicates and the overlapping distribution. In addition, genes with a low level of expression tend to have a large variance while genes highly expressed have a much smaller variance. Estimation of the correct fold changes especially for low level expressed genes with few replicates and large variability is difficult to precisely assess. Consequently, DESeq2 may not identify some of the genes potentially differentially expressed in our experiments.

To evaluate our data without shrinking effects, we thus next analyzed the RNAseq data for alpha and beta cells using the EdgeR method following the GC content and RIN corrections as applied for DESeq2. We identified for Venus+ alpha cells an extended list of 39 differentially expressed genes between LFD and HFD mice (Table 3). Most of these genes were upregulated (33/39) and corresponded to genes preferentially expressed in the exocrine tissue or in non-alpha cells, suggesting potential dedifferentiation or contamination. We indeed observed that genes coding for Trypsin (*Try4/5 and Prss2*), RNase (*RNase1*), Carboxypeptidase (*Cpa1*) and Elastase (*Cela3b*), all specific to acinar cells, were upregulated in the Venus+ sorted alpha cell fraction of HFD compared to LFD mice. Insulin genes *(Ins1/2)* and beta-cell expressed markers, proconvertase *Pcsk1* and islet amyloid polypeptide *Iapp*, were also increased whereas the delta-cell markers, somatostatin (*Sst*) and *Hhex*, were down-regulated in Venus+ sorted alpha cells from HFD mice. We also found that immune or hematopoietic cell markers (*Cd24a*, *Plvap*) were altered in HFD hyperglycemic mice. Importantly, RNA seq data revealed that some of the genes enriched in exocrine cells were barely detected in alpha cells from LFD mice (S1 File). Of note, *Reg2* was the most differentially regulated gene in alpha cells (HFD vs LFD: 39.39-fold).

**Table 3:** Differential gene expression analysis between HFD and LFD mice for pancreatic alpha cells using EdgeR.

Gene	Fold Regulation	Adjusted p-value
Reg2	39.39	8.55E-18
Try5	14.1	8.49E-07
Spink3	11.09	5.41E-05
Cela3b	10.42	7.62E-05
Prss2	8.64	1.78E-04
2210010C04Rik	8.24	9.29E-04
Clps	7.95	2.67E-05
Ctrb1	7.7	4.58E-04
Try4	7.63	4.11E-04
Pnlip	7.54	8.76E-04
Sycn	7.34	5.03E-03
Cpb1	7.26	3.21E-03
Cpa1	7.24	1.63E-03
Zg16	6.78	4.39E-08
Ctrl	6.06	6.19E-05
Pnliprp1	6.06	1.57E-03
Cela1	5.55	7.62E-05
Reg1	5.29	3.35E-03
Cela2a	4.88	2.36E-02
Ins1	4.83	1.31E-07
Rnase1	4.56	4.07E-03
Ctrc	4.33	8.28E-03
Ins2	4.15	4.65E-05
Pcsk1	3.93	2.58E-02
Krt19	3.83	2.46E-04
Nupr1	3.81	2.36E-02
Bace2	3.55	2.24E-04
Plvap	3.3	8.76E-04
Mgst1	2.75	8.76E-04
Upk3a	2.59	9.12E-03
G6pc2	2.47	3.49E-02
Iapp	2.01	2.34E-03
Adcy1	1.78	1.29E-02
Dpp6	-1.73	2.96E-02
Cd24a	-1.81	3.59E-02
Sst	-1.96	9.01E-03
Hhex	-1.98	6.51E-03
Snord14e	-2.2	4.67E-03
Acp1	-2.74	2.82E-02

mRNA levels from LFD and HFD Venus+ alpha cells were evaluated with RNA sequencing using EdgeR analyses (Covariates: GC mean, RIN; Adjusted p value ≤ 0.05). The fold regulation values represent the ratio of the results obtained for fold change between HFD to LFD mice.

Comparative analyses of Venus+ alpha cells from RNAseq data with DESeq2 and EdgeR methods shared eight genes differentially expressed (*Hhex*, *Krt19*, *Mgst1*, *Plvap*, *Sst*, *Dpp6*, *Iapp* and *Bace2*). In addition to the upregulation of several non-alpha cell markers, we also found that the 6 genes *Upk3a*, *8430408G22Rik* (*Depp1)*, *Mgst1*, *Syt5*, *Dpp6* or *Adcy1*, which were identified by one of both methodologies, were differentially expressed in alpha cells from HFD compared to LFD mice. We observed significant increases of *Upk3a*, *Syt5*, *C10orf10*, *Adcy1* and *Mgst1* and decreases of *Dpp6* mRNA levels. These genes, expressed at similar or higher levels in alpha cells compared to beta cells (S1 File), code for proteins involved in functional pathways including exocytosis (*Syt5*, *Dpp6*), autophagy (*Depp1*) and cellular stress defense (*Mgst1*, *Adcy1* and *Upk3a*). These observations thus indicate that HFD may induce changes on molecular components involved in alpha-cell function and potentially dedifferentiation.

Surprisingly, beta cell analyses (HFD vs LFD) taking the EdgeR method revealed only 99 genes differentially regulated compared to the 3192 identified genes with DESeq2 (Table 4). Among them, the four most highly differentially regulated genes code for proteins involved in proliferation (*Cdc20*, *Top2a*, *Ccnb2* and *Cdk1*) with expression levels superior to 20-fold in beta cells from HFD mice compared to controls.

**Table 4:** Differential gene expression analysis between HFD and LFD mice for pancreatic beta cells using EdgeR.

Gene name	Fold Regulation	Adjusted p-value
Cdc20	39.04	3.14E-17
Top2a	26.06	2.53E-10
Ccnb2	23.36	3.68E-10
Cdk1	22.48	7.36E-07
1190002F15Rik	14.64	6.16E-07
Pbk	13.91	1.48E-04
Gm11223	12.8	2.81E-06
Aldh1a3	10.26	5.61E-05
Cdca3	9.52	1.03E-06
Gc	8.76	1.21E-21
…	…	…
Lrrc16b	-5.27	8.62E-03
Crhr1	-5.39	8.62E-03
Emid1	-5.74	2.14E-02
Kcnj12	-6.32	3.01E-05
T2	-6.91	6.06E-04
D430019H16Rik	-8.37	3.67E-04
Clps	-25.44	1.90E-02
Cela1	-26.05	3.82E-02
Prss2	-34.63	4.08E-02
Sycn	-36.63	1.25E-03

mRNA levels from LFD and HFD Cherry+ beta cells were evaluated with RNA sequencing using EdgeR analyses (Covariates: GC mean, RIN; Adjusted p value ≤ 0.05). The fold regulation values represent the ratio of the results obtained from fold change between HFD to LFD mice. Only the 10 most up- and down-regulated genes are listed (among 99 differentially expressed genes).

### Gene ontology analyses

Gene ontology from differential analysis of RNAseq data for alpha cells in DIO mice highlighted an enrichment of genes coding for protein involved in pancreatic digestion (protein and lipid) and maturity onset diabetes of the young biological processes (KEGG and GO analysis) due to the presence of exocrine and beta-cell enriched marker genes (S3 Table). For beta cells, the GO/KEGG analyses indicate enrichment of genes coding for proteins involved in cell cycle biological processes, but also ion channel activities for cell function (S4 Table), in agreement with the proliferative state of beta cells as well as the alterations of insulin secretion previously described in HFD mice [24, 35].

### Validation of differentially expressed genes in Venus+ alpha cells in HFD hyperglycemic mice

**Fig 1:**
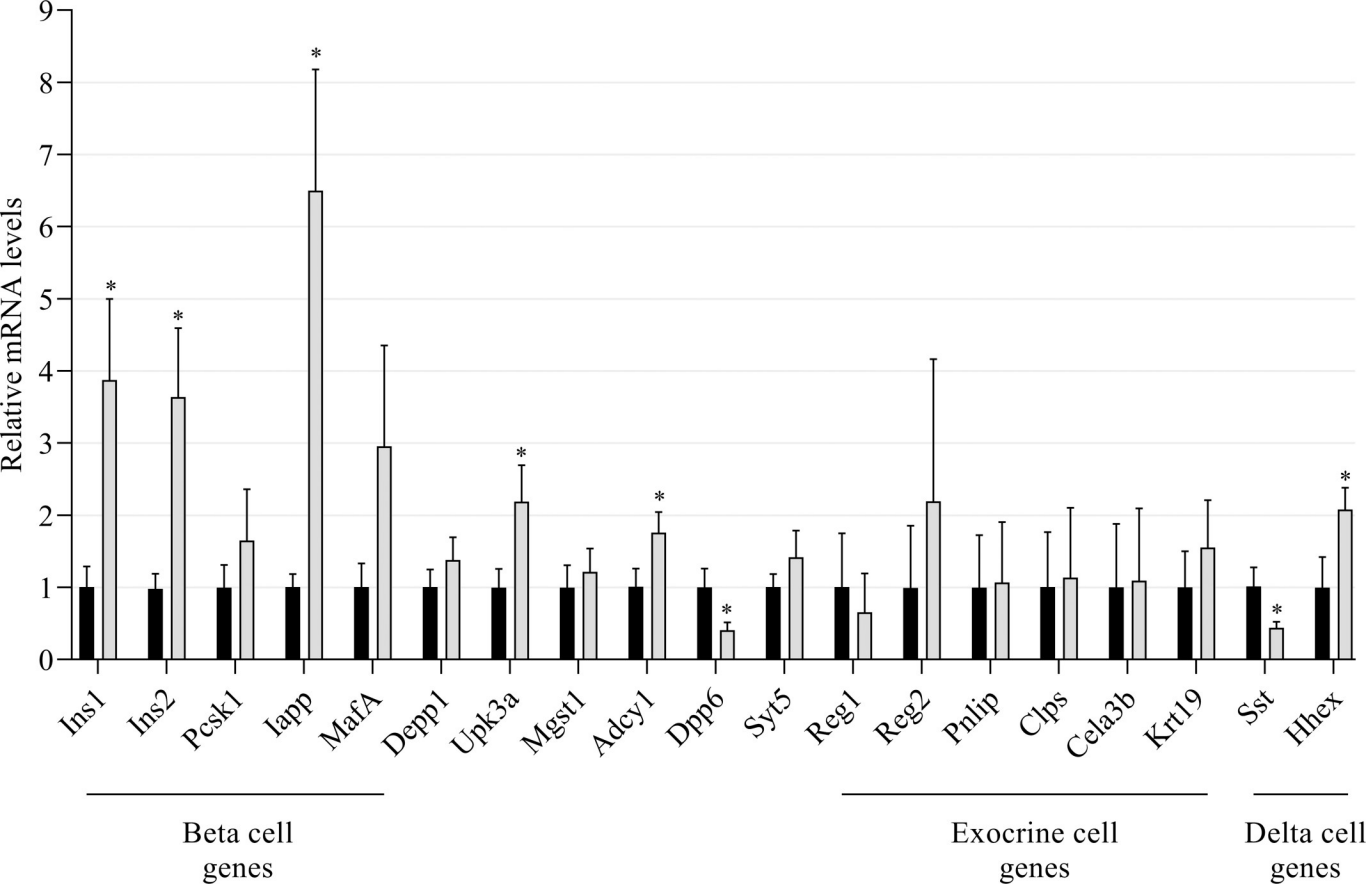
Validation of RNAseq results in DIO alpha-cell through real-time quantitative PCR analyses. FACS-sorted Venus+ alpha cells from control LFD (black bars) and obese hyperglycemic HFD (grey bars) mice were collected and analysed for mRNA quantification of altered genes identified by RNAseq. The measurement of different mRNA levels of target genes was performed by real-time PCR (Light-cycler LC480). Data are relative to three housekeeping genes (*Rps9*, *Hprt* and *Ppia*) and presented as the means (Fold of controls) ± SEM (n = 7 mice for LFD, n = 6 for HFD group). * means significant compared to LFD.

To further confirm the results of differentially expressed genes using the DESeq2 and EdgeR methods, we investigated their mRNA expression levels through qRT-PCR using new batches of sorted Venus+ alpha cells from LFD and HFD mice (Fig 1). First, we assessed expression of the potential functional target genes *Depp1*, *Upk3a*, *Mgst1*, *Adcy1*, *Dpp6*, *Syt5* in sorted alpha cells form LFD and HFD mice. We found that only the *Upk3a*, *Adcy1* and *Dpp6* genes were differentially expressed in the new collected samples of DIO mice whereas the *Depp1*, *Mgst1* and *Syt5* genes exhibited non-significant variations between HFD and LFD mice.

We then evaluated whether the identified genes, for exocrine (*Reg1*and *Reg2*, *Pnlip*, *Clps*, *Cela3b and Krt19*), beta (*Ins1*, *Ins2*, *Pcsk1/3*, *Iapp*, *MafA)* or delta *(Sst*, *Hhex)* cells markers, in HFD compared to LFD mice were detected at mRNA levels in sorted alpha cells by qRT-PCR. From these genes, only *Ins1*, *Ins2* and *Iapp* (beta cell genes) and *Sst* (delta cell gene) mRNA levels were confirmed to be regulated in HFD mice compared to controls. Of note, *Pcsk1/3* and *MafA* mRNA levels were slightly upregulated in alpha cells of HFD compared to LFD mice but without statistical significance.

In addition, the upregulation of exocrine cell marker genes found by RNAseq was not confirmed by qRT-PCR, illustrating the critical importance of multiple techniques to validate gene expression levels. To further verify the expression levels of exocrine- and beta-cell genes in Venus+ alpha cells in HFD hyperglycemic mice in alpha cells, we performed immunofluorescence experiments using pancreatic sections from LFD and HFD mice. We thus assessed the colocalisation between Venus and pancreatic lipase (PNLIP) or insulin in pancreatic islets (S2 Fig) and observed no relevant coexpression between these markers and Venus protein. This suggests that these proteins are not expressed in alpha cells, that the level of detection is too low, that mRNAs are not translated or that it might be an artefact from exocrine and beta-cell contaminations.

Taken together, our results indicate that enrichment of exocrine markers in alpha cells during HFD feeding is due to acinar and/or ductal cell contamination rather than potential variation of cell fate or transformation, while the increase in beta cell marker gene expression is either not accompanied by protein translation or also due to beta cell contamination.

Taken together, our study highlight that alpha cell gene expression is not quantitatively highly reactive to HFD feeding with only a few genes identified in cellular stress and exocytosis, which may contribute to alpha-cell alterations/adaptations in response to high-fat diet. The precise role of these targets will have to be further studied. By contrast, beta cells are markedly affected by HFD diet leading to major alterations of genes coding for proteins involved in secretion and proliferation processes.

Along with our previously reported genes, differentially regulated between HFD and LFD and coding for proteins involved in glucagon gene transcription and secretion, we conclude that alpha cell transcriptome is not strongly influenced by HFD.

## Discussion

Characterization of endocrine-cell functions and their transcriptomes in diabetes is critical to better understand how and by which mechanisms alpha and beta cells may dysfunction.

Single-cell RNAseq studies have emerged as new promising techniques to determine the molecular alterations of alpha- and beta-cell functions in diabetes [36]. However, except for the determination of cell-specific gene markers, data have not been reproducible especially taking results from single cell transcriptome analyses; we thus need to exert caution in the interpretation of the results. Among different reasons to explain conflicting results, time-lapse between pancreas harvesting and cell sorting including recovery periods and medium composition as well as islets sources may represent crucial points, especially in human studies. Indeed, recent studies clearly showed that ex vivo cultures of primary human endocrine cells led to dedifferentiation and expression of markers attributed to progenitor cells [37]. In addition, the type of treatment for diabetes, individual heterogeneity (sex, age, ethnic origin and death cause) as well as technical limitations (Fluidigm cell capture), cell exclusion criteria (multihormonal content cells) and methods of analysis also represent reasons for discrepant results. All these technical limitations lead to biases in the interpretation of RNAseq data, compromising proper molecular characterizations of alpha and beta cells in diabetes [38].

We thus chose a rodent diabetic model using transgenic mice, which exhibit specific labelling of glucagon- and insulin-producing cells. We acknowledge that DIO mice are closer to the prediabetic state as reported [24] and that our study is limited to male mice. Nevertheless, we were able to collect almost pure fractions of alpha and beta cells without recovery periods and analyze the effects of high-fat diet on the whole population of pancreatic endocrine cells without exclusion criteria.

We previously reported that sorted alpha cells from DIO mice exhibited partial functional and molecular alterations including moderate increases of *Gcg*, *Pou3f4*, *Pcsk1/3*, *Foxa1*, *cMaf*, *Scn3a* and *Cacna1c* mRNA levels in HFD mice among 50 candidate genes analyzed [24]. We now report that evaluation of the alpha cell transcriptome through comparative analysis between control LFD and obese hyperglycemic HFD mice allowed us to identify only three new differentially expressed targets (*Adcy1*, *Dpp6* and *Upk3a*). Although these three targets are expressed at substantial levels in alpha cells and at higher levels compared to beta cells, no role has been assigned to them in pancreatic endocrine cells.

*Adcy1* which code for one of eight isoforms of adenylate cyclase, activated by the calcium/calmodulin complex and mainly expressed in brain [39], could represent an activator of cell survival signaling pathways [40]. Furthermore, structural bladder membrane protein Uroplakin3a (*Upk3a*) was previously described as a marker of epithelial progenitor cells in response to post-injury repair associated with neuroendocrine hyperplasia [41]. This potentially suggests that upregulation of *Adcy1* and *Upk3a* gene expression in alpha cells may reflect molecular adaptations to HFD stress to prevent apoptosis, reinforcing the hypothesis that alpha cells are resistant to metabolic stress as previously described [42]. The *Dpp6* gene, which encodes for a transmembrane protein, was initially described to control Kv4 channel localization and activity [43, 44], and thus potentially take part in exocytosis. Interestingly, DPP6, which is widely expressed in the central nervous system, was recently identified as an endocrine cell marker especially in pancreatic alpha and beta cells [45]. To our knowledge, no direct link between these targets and diabetes or obesity have been described; thus further studies directed to the analysis of the specific roles of these three components would be of interest in diabetes.

Of note, the genes we have previously reported as differentially expressed between alpha cells from LFD and HFD mice, by a candidate gene approach [24], were not identified by our new analyses. A potential explanation could be that the expression levels of the mRNAs do not allow to obtain confident results through RNAseq technology. Since glucagon gene (*Gcg*) accounts for almost 1/3 of all reads in alpha cells, it is therefore more difficult to have enough reads in lowly expressed genes to call them differentially expressed. Indeed *Foxa1* and *cMaf* genes, which are expressed at very low levels in alpha cells, were not considered through DESeq2 or EdgeR analyses. The prohormone convertase *Pcsk1* gene, coding for protein involved in the maturation of proglucagon in GLP-1, was the only one to be confirmed by RNAseq analysis, although qPCR validation revealed a non-significant increase of its mRNA levels in alpha cells of obese mice compared to controls. It is thus likely that other targets expressed at either high or low levels could be differentially regulated between alpha cells from HFD and LFD mice.

Compared with alpha cells, beta cells are massively affected by HFD with a large number of genes differentially expressed between control and obese hyperglycemic mice. Our analyses revealed significant differences in the expression of genes coding for proteins involved in cell cycle and proliferation, ion channel activities and exocytosis processes as it was previously reported [34, 46]. EdgeR analysis revealed robust variations for genes associated to cell cycle and proliferation such as *Cdc20*, *Top2a*, *Ccnb2* and *Cdk1*. Furthermore, the highest upregulated gene in DESeq2 analysis was the *Gc* gene which codes for a protein involved in the control of beta cell mass in agreement to beta cell hyperplasia observed in DIO mice [24, 35]. Interestingly, we observed decreased expression of several genes coding for proteins involved in potentiation of GSIS (*T2* and *Trpm5*) as well as increase of *Rgs4* gene coding for a protein associated to inhibition of GSIS, all suggesting functional defects of beta cell secretion in response to glucose as we previously described [24].

As described in multiple studies, endocrine cell identity can be modified during diabetes [23, 47–49]. Importantly single-cell technologies have revealed that mature endocrine alpha and beta cells express low levels of specific mRNAs enriched in other endocrine or exocrine cell types [25, 50] although this observation is controversial, as it has been suggested to reflect potential artefacts such as contamination by other cell types [38]. Although sorted alpha cells selectively expressed *Gcg* and *Pou3f4* and are highly enriched for the expression of *Arx*, *MafB* but also Irx2 and *Ttr* genes as previously reported [26, 38], our data also indicate that sorted alpha cells express low amounts of mRNA corresponding to beta, delta and exocrine cell markers. Indeed, variation of *Ins1*, *Ins2 and Iapp* gene expression were validated by real-time qPCR suggesting that beta cell markers could be either induced in alpha cells from HFD mice or due to beta cell contamination. Interestingly the relative levels of *Ins1*, *Ins2*, *MafA a*nd *Iapp* genes in sorted beta cells were not different between LFD and HFD mice, suggesting that minor beta cell contamination of alpha cell fractions might be involved with higher number of beta cells contaminating alpha cells in HFD mice, characterized by beta-cell hyperplasia [24]. In addition, we could not detect the insulin protein by immunofluorescence staining in pancreatic alpha cells both in LFD and HFD mice, confirming the lack of functional relevance of these observations.

We also identified increases of genes coding for exocrine cell markers in alpha cells from HFD mice such as Pnlip/Clps and Reg1/Reg2 mRNAs before qPCR validation. Assessment by qRT-PCR however invalidated the RNAseq results; furthermore, we did not detect their encoded protein indicating that these findings were due to minor contamination of exocrine cells, and not to potential dedifferentiation of alpha cells, as described in insulinopenic hyperglycemic mouse models [51]. Thus, our results do not support potential dedifferentiation or transdifferentiation of alpha cells into pluripotent cells, which express alpha and non-alpha cell markers, at least in our model.

In conclusion, the transcriptome of alpha cells is not highly affected by HFD in obese hyperglycemic mice as only a minor fraction of the genes analyzed was found to be altered compared to LFD mice and the fold difference in the regulated genes was relatively small. This is in marked contrast to beta cells of HFD obese hyperglycemic mice. The alpha cell might be thus less sensitive to metabolic stresses compared to beta cells and thus more resistant to dysfunction and death.

## Supporting information

S1 Checklist ARRIVE guidelines checklist. (PDF)

S1 Fig Schematic representation of experimental procedures. (PPTX)

S2 Fig Immunodetection of PNLIP, insulin and Venus on pancreatic sections from obese HFD mice. (PPTX)

S1 File Deseq2 analysis of LFD samples_beta vs alpha. Differential gene expression analysis between beta- and alpha-cell transcriptomes in control LFD group using Deseq2.
(TSV)

S2 File Deseq2 analysis of alpha cells_HFD vs LFD. Differential gene expression analysis between HFD and LFD groups for alpha-cell transcriptome using Deseq2.
(TSV)

S3 File Deseq2 analysis of beta cells_HFD vs LFD. Differential gene expression analysis between HFD and LFD groups for beta-cell transcriptome using Deseq2.
(TSV)

S4 File EdgeR analysis of alpha cells_HFD vs LFD. Differential gene expression analysis between HFD and LFD groups for alpha-cell transcriptome using EdgeR.
(TSV)

S5 File EdgeR analysis of beta cells_HFD vs LFD. Differential gene expression analysis between HFD and LFD groups for beta-cell transcriptome using EdgeR.
(TSV)

S1 Table Diet-fed mice characteristics. (PPTX)

S2 Table Differential gene expression analysis between alpha and beta cell types for LFD mice using DESeq2. (PPTX)

S3 Table Gene ontology from differential expression analysis of RNAseq data for Venus+ alpha cells in HFD mice. (PPTX)

S4 Table Gene ontology from differential gene expression analysis of RNAseq data for Cherry+ beta cells in HFD mice. (PPTX)

## Abbreviations

HbA1c: glycated hemoglobin (%)
RPS9: ribosomic protein 9S
SEM: Standard Error of the Mean (= Standard Deviation/√(sample size))
NEFA: non-esterified fatty-acids
