# Supplementary material for: High-fat diet impacts more changes in beta-cell compared to alpha-cell transcriptome
Source: PLoS One. 2019 Mar 8;14(3):e0213299. doi: 10.1371/journal.pone.0213299 (PMC6407777; doi:10.1371/journal.pone.0213299)
Supplement: S1 Fig — (PPTX) [file pone.0213299.s002.pptx]

## Slide 1
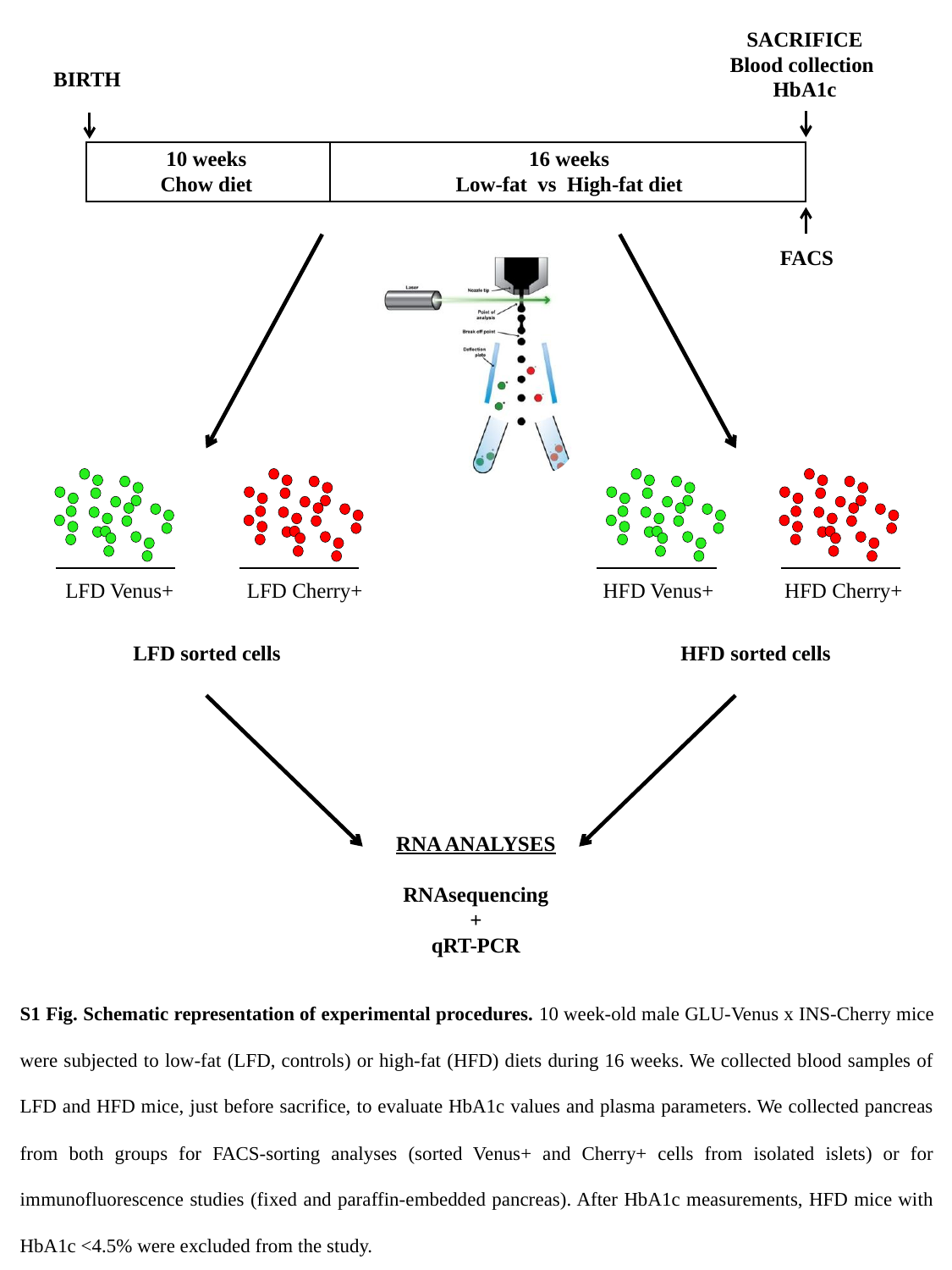

SACRIFICE
Blood collection
HbA1c
BIRTH
10 weeks
Chow diet
16 weeks
Low-fat vs High-fat diet
FACS
HFD Cherry+
LFD Venus+
LFD Cherry+
HFD Venus+
LFD sorted cells
HFD sorted cells
RNA ANALYSES
RNAsequencing
+
qRT-PCR
S1 Fig. Schematic representation of experimental procedures. 10 week-old male GLU-Venus x INS-Cherry mice were subjected to low-fat (LFD, controls) or high-fat (HFD) diets during 16 weeks. We collected blood samples of LFD and HFD mice, just before sacrifice, to evaluate HbA1c values and plasma parameters. We collected pancreas from both groups for FACS-sorting analyses (sorted Venus+ and Cherry+ cells from isolated islets) or for immunofluorescence studies (fixed and paraffin-embedded pancreas). After HbA1c measurements, HFD mice with HbA1c <4.5% were excluded from the study.
