# Supplementary material for: High-fat diet impacts more changes in beta-cell compared to alpha-cell transcriptome
Source: PLoS One. 2019 Mar 8;14(3):e0213299. doi: 10.1371/journal.pone.0213299 (PMC6407777; doi:10.1371/journal.pone.0213299)
Supplement: S2 Fig — (PPTX) [file pone.0213299.s003.pptx]

## Slide 1
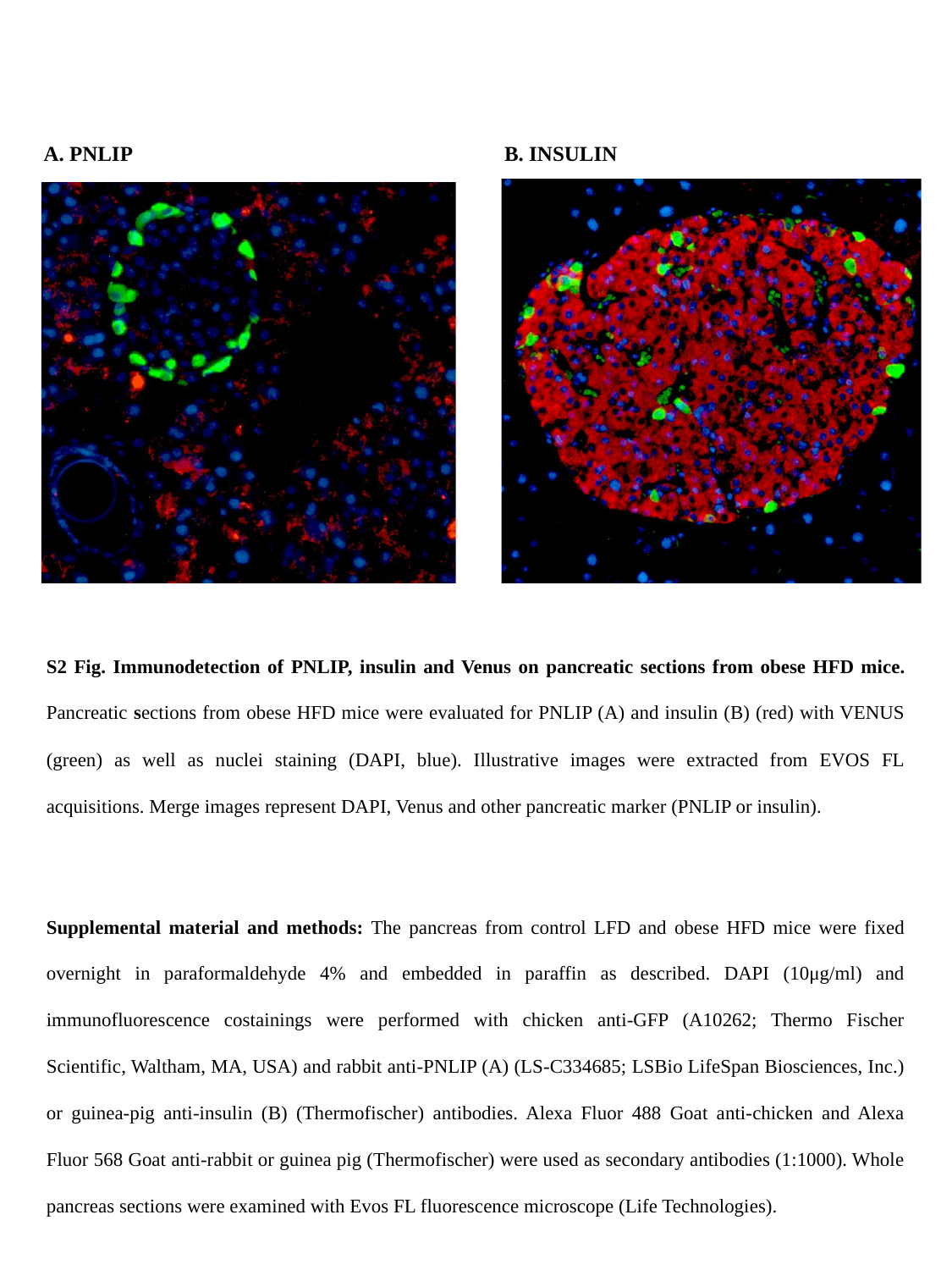

B. INSULIN
A. PNLIP
S2 Fig. Immunodetection of PNLIP, insulin and Venus on pancreatic sections from obese HFD mice. Pancreatic sections from obese HFD mice were evaluated for PNLIP (A) and insulin (B) (red) with VENUS (green) as well as nuclei staining (DAPI, blue). Illustrative images were extracted from EVOS FL acquisitions. Merge images represent DAPI, Venus and other pancreatic marker (PNLIP or insulin).
Supplemental material and methods: The pancreas from control LFD and obese HFD mice were fixed overnight in paraformaldehyde 4% and embedded in paraffin as described. DAPI (10μg/ml) and immunofluorescence costainings were performed with chicken anti-GFP (A10262; Thermo Fischer Scientific, Waltham, MA, USA) and rabbit anti-PNLIP (A) (LS-C334685; LSBio LifeSpan Biosciences, Inc.) or guinea-pig anti-insulin (B) (Thermofischer) antibodies. Alexa Fluor 488 Goat anti-chicken and Alexa Fluor 568 Goat anti-rabbit or guinea pig (Thermofischer) were used as secondary antibodies (1:1000). Whole pancreas sections were examined with Evos FL fluorescence microscope (Life Technologies).
