# Supplementary material for: High-fat diet impacts more changes in beta-cell compared to alpha-cell transcriptome
Source: PLoS One. 2019 Mar 8;14(3):e0213299. doi: 10.1371/journal.pone.0213299 (PMC6407777; doi:10.1371/journal.pone.0213299)
Supplement: S1 Table — (PPTX) [file pone.0213299.s009.pptx]

## Slide 1
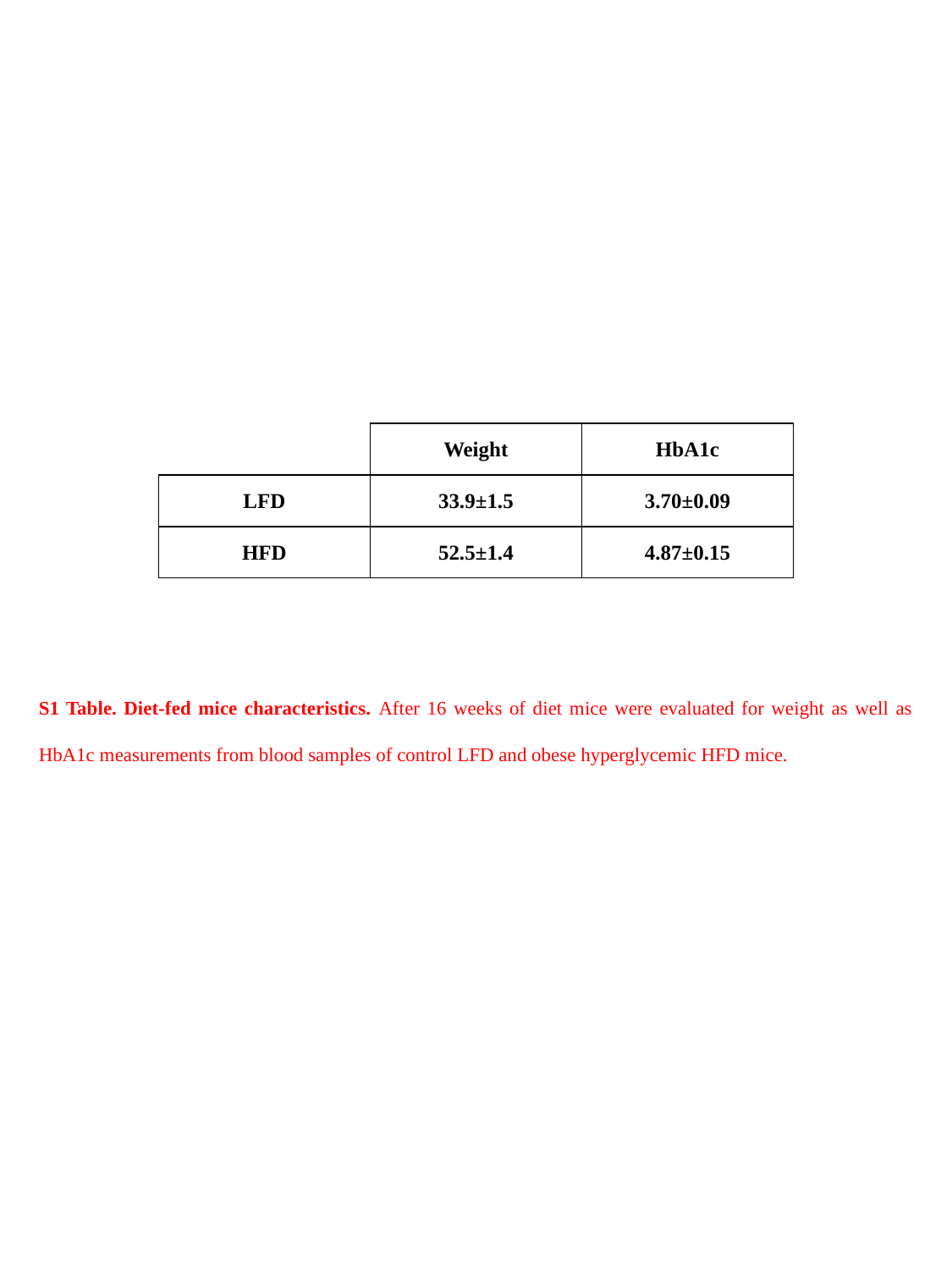

| | Weight | HbA1c |
| --- | --- | --- |
| LFD | 33.9±1.5 | 3.70±0.09 |
| HFD | 52.5±1.4 | 4.87±0.15 |
S1 Table. Diet-fed mice characteristics. After 16 weeks of diet mice were evaluated for weight as well as HbA1c measurements from blood samples of control LFD and obese hyperglycemic HFD mice.
