# Supplementary material for: High-fat diet impacts more changes in beta-cell compared to alpha-cell transcriptome
Source: PLoS One. 2019 Mar 8;14(3):e0213299. doi: 10.1371/journal.pone.0213299 (PMC6407777; doi:10.1371/journal.pone.0213299)
Supplement: S2 Table — (PPTX) [file pone.0213299.s010.pptx]

## Slide 1
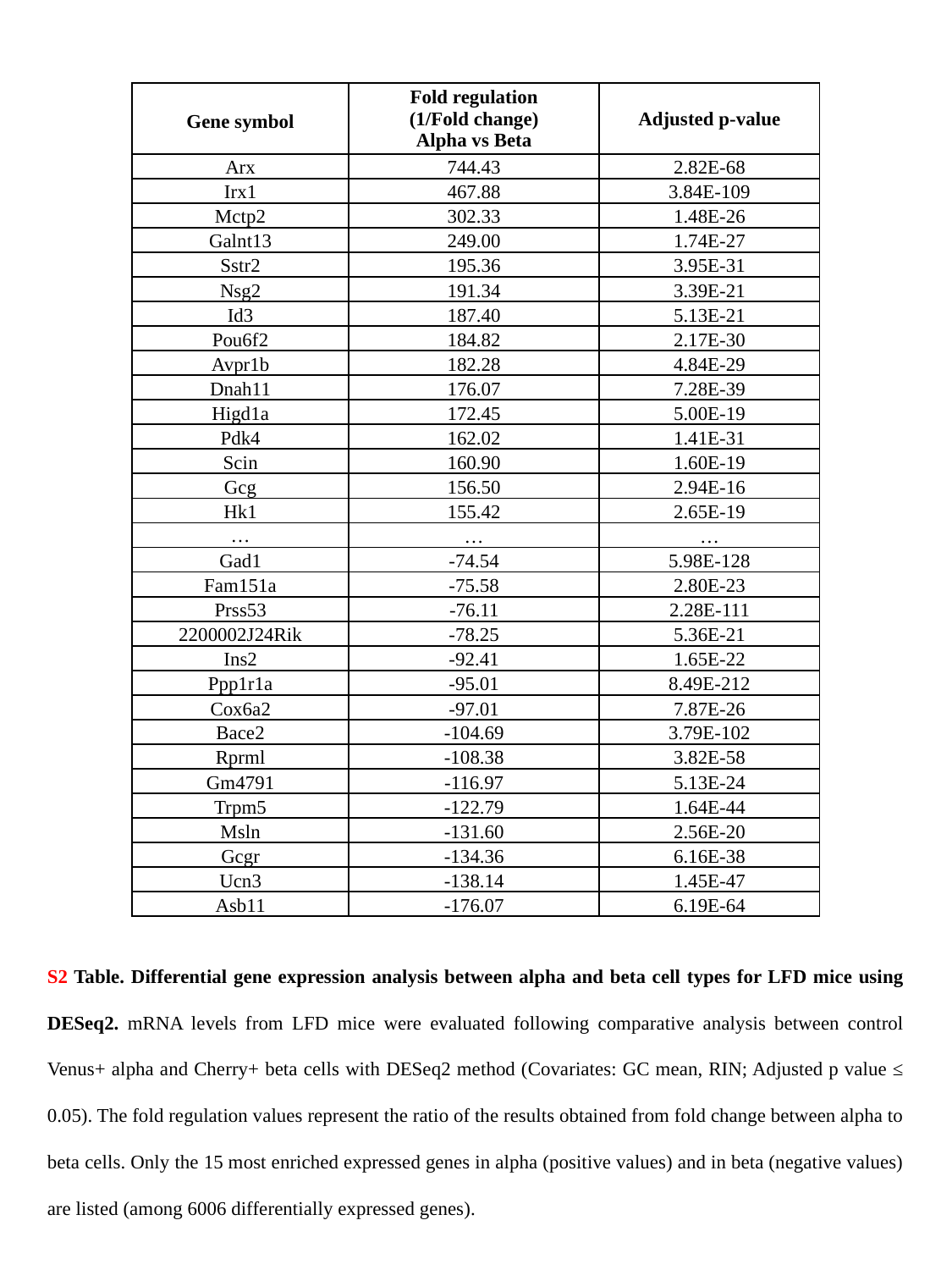

| Gene symbol | Fold regulation (1/Fold change) Alpha vs Beta | Adjusted p-value |
| --- | --- | --- |
| Arx | 744.43 | 2.82E-68 |
| Irx1 | 467.88 | 3.84E-109 |
| Mctp2 | 302.33 | 1.48E-26 |
| Galnt13 | 249.00 | 1.74E-27 |
| Sstr2 | 195.36 | 3.95E-31 |
| Nsg2 | 191.34 | 3.39E-21 |
| Id3 | 187.40 | 5.13E-21 |
| Pou6f2 | 184.82 | 2.17E-30 |
| Avpr1b | 182.28 | 4.84E-29 |
| Dnah11 | 176.07 | 7.28E-39 |
| Higd1a | 172.45 | 5.00E-19 |
| Pdk4 | 162.02 | 1.41E-31 |
| Scin | 160.90 | 1.60E-19 |
| Gcg | 156.50 | 2.94E-16 |
| Hk1 | 155.42 | 2.65E-19 |
| … | … | … |
| Gad1 | -74.54 | 5.98E-128 |
| Fam151a | -75.58 | 2.80E-23 |
| Prss53 | -76.11 | 2.28E-111 |
| 2200002J24Rik | -78.25 | 5.36E-21 |
| Ins2 | -92.41 | 1.65E-22 |
| Ppp1r1a | -95.01 | 8.49E-212 |
| Cox6a2 | -97.01 | 7.87E-26 |
| Bace2 | -104.69 | 3.79E-102 |
| Rprml | -108.38 | 3.82E-58 |
| Gm4791 | -116.97 | 5.13E-24 |
| Trpm5 | -122.79 | 1.64E-44 |
| Msln | -131.60 | 2.56E-20 |
| Gcgr | -134.36 | 6.16E-38 |
| Ucn3 | -138.14 | 1.45E-47 |
| Asb11 | -176.07 | 6.19E-64 |
S2 Table. Differential gene expression analysis between alpha and beta cell types for LFD mice using DESeq2. mRNA levels from LFD mice were evaluated following comparative analysis between control Venus+ alpha and Cherry+ beta cells with DESeq2 method (Covariates: GC mean, RIN; Adjusted p value ≤ 0.05). The fold regulation values represent the ratio of the results obtained from fold change between alpha to beta cells. Only the 15 most enriched expressed genes in alpha (positive values) and in beta (negative values) are listed (among 6006 differentially expressed genes).
