# Supplementary material for: High-fat diet impacts more changes in beta-cell compared to alpha-cell transcriptome
Source: PLoS One. 2019 Mar 8;14(3):e0213299. doi: 10.1371/journal.pone.0213299 (PMC6407777; doi:10.1371/journal.pone.0213299)
Supplement: S3 Table — (PPTX) [file pone.0213299.s011.pptx]

## Slide 1
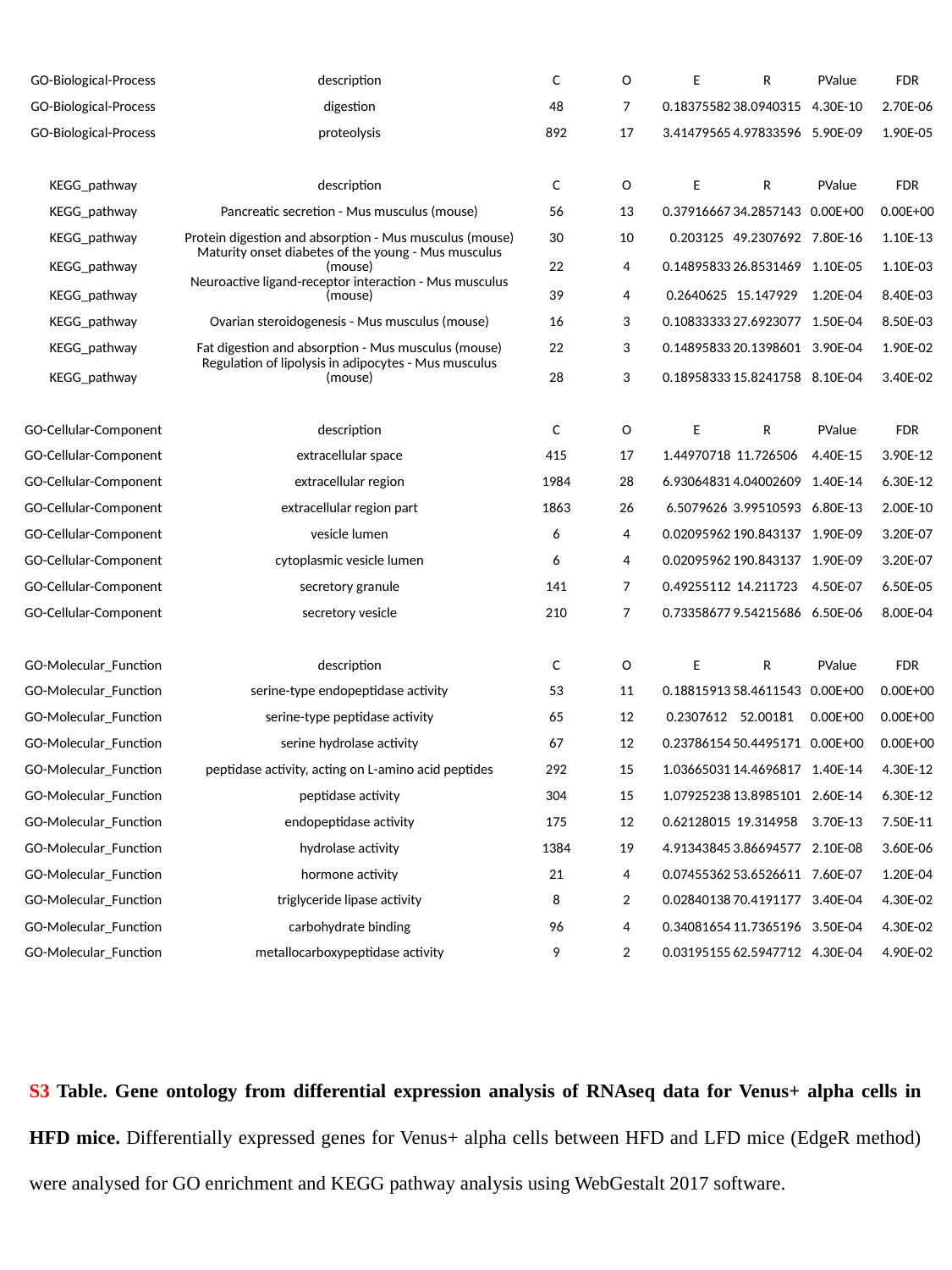

| GO-Biological-Process | description | C | O | E | R | PValue | FDR |
| --- | --- | --- | --- | --- | --- | --- | --- |
| GO-Biological-Process | digestion | 48 | 7 | 0.18375582 | 38.0940315 | 4.30E-10 | 2.70E-06 |
| GO-Biological-Process | proteolysis | 892 | 17 | 3.41479565 | 4.97833596 | 5.90E-09 | 1.90E-05 |
| | | | | | | | |
| KEGG\_pathway | description | C | O | E | R | PValue | FDR |
| KEGG\_pathway | Pancreatic secretion - Mus musculus (mouse) | 56 | 13 | 0.37916667 | 34.2857143 | 0.00E+00 | 0.00E+00 |
| KEGG\_pathway | Protein digestion and absorption - Mus musculus (mouse) | 30 | 10 | 0.203125 | 49.2307692 | 7.80E-16 | 1.10E-13 |
| KEGG\_pathway | Maturity onset diabetes of the young - Mus musculus (mouse) | 22 | 4 | 0.14895833 | 26.8531469 | 1.10E-05 | 1.10E-03 |
| KEGG\_pathway | Neuroactive ligand-receptor interaction - Mus musculus (mouse) | 39 | 4 | 0.2640625 | 15.147929 | 1.20E-04 | 8.40E-03 |
| KEGG\_pathway | Ovarian steroidogenesis - Mus musculus (mouse) | 16 | 3 | 0.10833333 | 27.6923077 | 1.50E-04 | 8.50E-03 |
| KEGG\_pathway | Fat digestion and absorption - Mus musculus (mouse) | 22 | 3 | 0.14895833 | 20.1398601 | 3.90E-04 | 1.90E-02 |
| KEGG\_pathway | Regulation of lipolysis in adipocytes - Mus musculus (mouse) | 28 | 3 | 0.18958333 | 15.8241758 | 8.10E-04 | 3.40E-02 |
| | | | | | | | |
| GO-Cellular-Component | description | C | O | E | R | PValue | FDR |
| GO-Cellular-Component | extracellular space | 415 | 17 | 1.44970718 | 11.726506 | 4.40E-15 | 3.90E-12 |
| GO-Cellular-Component | extracellular region | 1984 | 28 | 6.93064831 | 4.04002609 | 1.40E-14 | 6.30E-12 |
| GO-Cellular-Component | extracellular region part | 1863 | 26 | 6.5079626 | 3.99510593 | 6.80E-13 | 2.00E-10 |
| GO-Cellular-Component | vesicle lumen | 6 | 4 | 0.02095962 | 190.843137 | 1.90E-09 | 3.20E-07 |
| GO-Cellular-Component | cytoplasmic vesicle lumen | 6 | 4 | 0.02095962 | 190.843137 | 1.90E-09 | 3.20E-07 |
| GO-Cellular-Component | secretory granule | 141 | 7 | 0.49255112 | 14.211723 | 4.50E-07 | 6.50E-05 |
| GO-Cellular-Component | secretory vesicle | 210 | 7 | 0.73358677 | 9.54215686 | 6.50E-06 | 8.00E-04 |
| | | | | | | | |
| GO-Molecular\_Function | description | C | O | E | R | PValue | FDR |
| GO-Molecular\_Function | serine-type endopeptidase activity | 53 | 11 | 0.18815913 | 58.4611543 | 0.00E+00 | 0.00E+00 |
| GO-Molecular\_Function | serine-type peptidase activity | 65 | 12 | 0.2307612 | 52.00181 | 0.00E+00 | 0.00E+00 |
| GO-Molecular\_Function | serine hydrolase activity | 67 | 12 | 0.23786154 | 50.4495171 | 0.00E+00 | 0.00E+00 |
| GO-Molecular\_Function | peptidase activity, acting on L-amino acid peptides | 292 | 15 | 1.03665031 | 14.4696817 | 1.40E-14 | 4.30E-12 |
| GO-Molecular\_Function | peptidase activity | 304 | 15 | 1.07925238 | 13.8985101 | 2.60E-14 | 6.30E-12 |
| GO-Molecular\_Function | endopeptidase activity | 175 | 12 | 0.62128015 | 19.314958 | 3.70E-13 | 7.50E-11 |
| GO-Molecular\_Function | hydrolase activity | 1384 | 19 | 4.91343845 | 3.86694577 | 2.10E-08 | 3.60E-06 |
| GO-Molecular\_Function | hormone activity | 21 | 4 | 0.07455362 | 53.6526611 | 7.60E-07 | 1.20E-04 |
| GO-Molecular\_Function | triglyceride lipase activity | 8 | 2 | 0.02840138 | 70.4191177 | 3.40E-04 | 4.30E-02 |
| GO-Molecular\_Function | carbohydrate binding | 96 | 4 | 0.34081654 | 11.7365196 | 3.50E-04 | 4.30E-02 |
| GO-Molecular\_Function | metallocarboxypeptidase activity | 9 | 2 | 0.03195155 | 62.5947712 | 4.30E-04 | 4.90E-02 |
S3 Table. Gene ontology from differential expression analysis of RNAseq data for Venus+ alpha cells in HFD mice. Differentially expressed genes for Venus+ alpha cells between HFD and LFD mice (EdgeR method) were analysed for GO enrichment and KEGG pathway analysis using WebGestalt 2017 software.
