# Supplementary material for: High-fat diet impacts more changes in beta-cell compared to alpha-cell transcriptome
Source: PLoS One. 2019 Mar 8;14(3):e0213299. doi: 10.1371/journal.pone.0213299 (PMC6407777; doi:10.1371/journal.pone.0213299)
Supplement: S4 Table — (PPTX) [file pone.0213299.s012.pptx]

## Slide 1
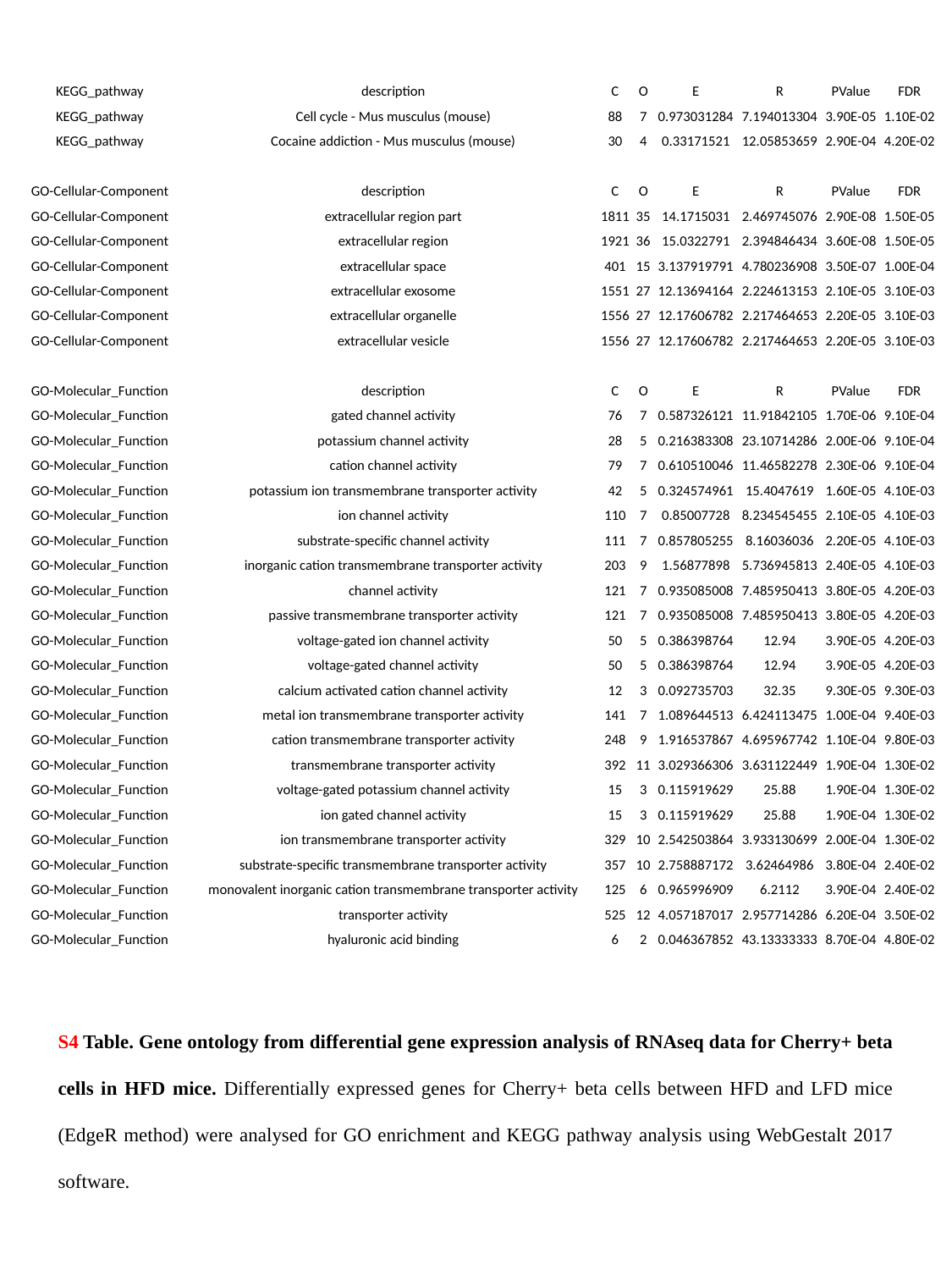

| KEGG\_pathway | description | C | O | E | R | PValue | FDR |
| --- | --- | --- | --- | --- | --- | --- | --- |
| KEGG\_pathway | Cell cycle - Mus musculus (mouse) | 88 | 7 | 0.973031284 | 7.194013304 | 3.90E-05 | 1.10E-02 |
| KEGG\_pathway | Cocaine addiction - Mus musculus (mouse) | 30 | 4 | 0.33171521 | 12.05853659 | 2.90E-04 | 4.20E-02 |
| | | | | | | | |
| GO-Cellular-Component | description | C | O | E | R | PValue | FDR |
| GO-Cellular-Component | extracellular region part | 1811 | 35 | 14.1715031 | 2.469745076 | 2.90E-08 | 1.50E-05 |
| GO-Cellular-Component | extracellular region | 1921 | 36 | 15.0322791 | 2.394846434 | 3.60E-08 | 1.50E-05 |
| GO-Cellular-Component | extracellular space | 401 | 15 | 3.137919791 | 4.780236908 | 3.50E-07 | 1.00E-04 |
| GO-Cellular-Component | extracellular exosome | 1551 | 27 | 12.13694164 | 2.224613153 | 2.10E-05 | 3.10E-03 |
| GO-Cellular-Component | extracellular organelle | 1556 | 27 | 12.17606782 | 2.217464653 | 2.20E-05 | 3.10E-03 |
| GO-Cellular-Component | extracellular vesicle | 1556 | 27 | 12.17606782 | 2.217464653 | 2.20E-05 | 3.10E-03 |
| | | | | | | | |
| GO-Molecular\_Function | description | C | O | E | R | PValue | FDR |
| GO-Molecular\_Function | gated channel activity | 76 | 7 | 0.587326121 | 11.91842105 | 1.70E-06 | 9.10E-04 |
| GO-Molecular\_Function | potassium channel activity | 28 | 5 | 0.216383308 | 23.10714286 | 2.00E-06 | 9.10E-04 |
| GO-Molecular\_Function | cation channel activity | 79 | 7 | 0.610510046 | 11.46582278 | 2.30E-06 | 9.10E-04 |
| GO-Molecular\_Function | potassium ion transmembrane transporter activity | 42 | 5 | 0.324574961 | 15.4047619 | 1.60E-05 | 4.10E-03 |
| GO-Molecular\_Function | ion channel activity | 110 | 7 | 0.85007728 | 8.234545455 | 2.10E-05 | 4.10E-03 |
| GO-Molecular\_Function | substrate-specific channel activity | 111 | 7 | 0.857805255 | 8.16036036 | 2.20E-05 | 4.10E-03 |
| GO-Molecular\_Function | inorganic cation transmembrane transporter activity | 203 | 9 | 1.56877898 | 5.736945813 | 2.40E-05 | 4.10E-03 |
| GO-Molecular\_Function | channel activity | 121 | 7 | 0.935085008 | 7.485950413 | 3.80E-05 | 4.20E-03 |
| GO-Molecular\_Function | passive transmembrane transporter activity | 121 | 7 | 0.935085008 | 7.485950413 | 3.80E-05 | 4.20E-03 |
| GO-Molecular\_Function | voltage-gated ion channel activity | 50 | 5 | 0.386398764 | 12.94 | 3.90E-05 | 4.20E-03 |
| GO-Molecular\_Function | voltage-gated channel activity | 50 | 5 | 0.386398764 | 12.94 | 3.90E-05 | 4.20E-03 |
| GO-Molecular\_Function | calcium activated cation channel activity | 12 | 3 | 0.092735703 | 32.35 | 9.30E-05 | 9.30E-03 |
| GO-Molecular\_Function | metal ion transmembrane transporter activity | 141 | 7 | 1.089644513 | 6.424113475 | 1.00E-04 | 9.40E-03 |
| GO-Molecular\_Function | cation transmembrane transporter activity | 248 | 9 | 1.916537867 | 4.695967742 | 1.10E-04 | 9.80E-03 |
| GO-Molecular\_Function | transmembrane transporter activity | 392 | 11 | 3.029366306 | 3.631122449 | 1.90E-04 | 1.30E-02 |
| GO-Molecular\_Function | voltage-gated potassium channel activity | 15 | 3 | 0.115919629 | 25.88 | 1.90E-04 | 1.30E-02 |
| GO-Molecular\_Function | ion gated channel activity | 15 | 3 | 0.115919629 | 25.88 | 1.90E-04 | 1.30E-02 |
| GO-Molecular\_Function | ion transmembrane transporter activity | 329 | 10 | 2.542503864 | 3.933130699 | 2.00E-04 | 1.30E-02 |
| GO-Molecular\_Function | substrate-specific transmembrane transporter activity | 357 | 10 | 2.758887172 | 3.62464986 | 3.80E-04 | 2.40E-02 |
| GO-Molecular\_Function | monovalent inorganic cation transmembrane transporter activity | 125 | 6 | 0.965996909 | 6.2112 | 3.90E-04 | 2.40E-02 |
| GO-Molecular\_Function | transporter activity | 525 | 12 | 4.057187017 | 2.957714286 | 6.20E-04 | 3.50E-02 |
| GO-Molecular\_Function | hyaluronic acid binding | 6 | 2 | 0.046367852 | 43.13333333 | 8.70E-04 | 4.80E-02 |
S4 Table. Gene ontology from differential gene expression analysis of RNAseq data for Cherry+ beta cells in HFD mice. Differentially expressed genes for Cherry+ beta cells between HFD and LFD mice (EdgeR method) were analysed for GO enrichment and KEGG pathway analysis using WebGestalt 2017 software.
